# Immune Response to Conjugates of Fragments of the Type K9 Capsular Polysaccharide of Acinetobacter baumannii with Carrier Proteins

**DOI:** 10.1128/spectrum.01674-22

**Published:** 2022-08-18

**Authors:** Natalia Rudenko, Anna Karatovskaya, Anna Zamyatina, Anna Shepelyakovskaya, Svetlana Semushina, Fedor Brovko, Anna Shpirt, Vladimir Torgov, Natalia Kolotyrkina, Alexandr Zinin, Anastasiya Kasimova, Andrei Perepelov, Mikhail Shneider, Yuriy Knirel

**Affiliations:** a Laboratory of Immunochemistry, Pushchino Branch, Shemyakin-Ovchinnikov Institute of Bioorganic Chemistry, Russian Academy of Sciences, Pushchino, Moscow Region, Russia; b Laboratory of Carbohydrates and Biocides, N.D. Zelinsky Institute of Organic Chemistry, Russian Academy of Sciences, Moscow, Russia; c Laboratory of Molecular Bioengineering, Shemyakin-Ovchinnikov Institute of Bioorganic Chemistry, Russian Academy of Sciences, Moscow, Russia; Emory University School of Medicine

**Keywords:** *Acinetobacter baumannii*, capsular polysaccharide, interleukins, glycoconjugate, opsonisation assay

## Abstract

The clonal bacterial species Acinetobacter baumannii is an emerging multidrug-resistant pathogen which causes high-lethality infections. Cells of A. baumannii are surrounded by the type-specific capsular polysaccharide (CPS), which provides resistance to the protective mechanisms of the host and is considered a target for immunization. The conjugates of three inert carrier proteins and A. baumannii type K9 CPS fragments, which contained various numbers of oligosaccharide repeats (K-units), were synthesized by periodate oxidation and squaric acid chemistry. The conjugates were applied to immunize mice, and chemical synthesis by squaric acid was shown to significantly improve the immunogenic properties of glycoconjugate. In BALB/c mice, IgG antibodies were predominant among type K9 CPS reactive antibodies, and their total content was several times higher than that of IgM. Immune sera were characterized by their opsonization ability during practically the entire lives of the experimental mice. The sera were cross-reactive, but the highest specificity was observed against the antigen (type K9 CPS) used for immunization. The immunization of BALB/c and ICR-1 mice with a glycoconjugate without adjuvants led to varying degrees of stimulation of IL-10, IL-17A, and TNF-α production, but not IL-4 production in the ICR-1 mice. This is in contrast to the BALB/c mice, in which γ-IFN production was also activated. The protective effectiveness of the glycoconjugates obtained by squaric acid chemistry was demonstrated by experiments that involved challenging immunized and nonimmunized animals with a lethal dose of A. baumannii K9.

**IMPORTANCE** Immunization by glycoconjugates with A. baumannii type K9 CPS fragments induced a high level of antibodies (predominantly IgG) in sera, which reacted specifically with the CPS of A. baumannii type K9, as well as a long immunological memory. The sera of immunized animals efficiently opsonized A. baumannii type K9. Immunization resulted in the balanced production of pro/anti-inflammatory lymphokines and protective antibodies to ensure the survival of the mice infected with A. baumannii. The level of specific antibodies was sufficient to provide protective immunity against the challenge by A. baumannii, making this approach applicable in the development of vaccine preparations.

## INTRODUCTION

The Gram-negative bacterium Acinetobacter baumannii is a globally distributed, opportunistic pathogen that causes high-lethality nosocomial infections (1–7). Pulmonary symptoms of pneumonia caused by COVID-19 require the ventilation of the lungs, which increases bacterial coinfections of the respiratory tract, including those caused by A. baumannii (8–10). A. baumannii is characterized by multiple mechanisms of antibiotic resistance which, in some clinical isolates, may reach complete resistance.

Interest in A. baumannii virulence factors and the development of vaccines for protection against this bacterium are constantly increasing. One of the virulence factors of A. baumannii is the type-specific capsular polysaccharide (CPS, K-antigen).

СPSs are preferable vaccine candidates due to their occurrence on the cell surface, their immunogenicity, and their role in disease pathogenesis (11). Upon conjugation with an inert carrier protein, CPS is converted into a T-dependent antigen which can induce both B-cell and T-cell responses (12, 13).

The great health benefits of glycoconjugate vaccines in controlling bacterial diseases are due to the generation of a peptide-bound carbohydrate epitope on the surface of antigen-presenting cells for T-cell recognition (14).

Many variables in the design, development, and production of glycoconjugate vaccines affect their immunogenicity and, presumably, their efficacy. The choice of the saccharide size, carrier protein, conjugation chemistry, and formulation are some of the key points considered in every glycoconjugate development program. Recent contributions from glycoconjugate vaccines have been reported, featuring a rapid impact on diseases caused by a number of encapsulated bacteria, including Haemophilus influenzae type b, pneumococcus, and meningococcus (13, 15–20).

No licensed vaccines against pathogenic A. baumannii strains currently exist, and glycoconjugates based on capsular polysaccharides have not yet been described (21). In this work, we demonstrate the effectiveness of synthetic glycoconjugates based on A. baumannii CPS fragments as immunogens; that is, we present evidence to support prototypes of vaccines that induce antibodies against infections caused by A. baumannii.

## RESULTS

### Chemical synthesis of glycoconjugates.

A strain that produced K9 CPS was chosen for the synthesis. The chemical structure of this CPS was established in 1996 (22, 23). Some other strains with K9 CPS structure were isolated from another hospital source in 2020 and were shown to be similar (24). The K9 CPS structures from other strains had been invariant for 14 years, so we decided to prepare glycoconjugates based on K9 CPS.

The glycoconjugates were prepared by the partial periodate oxidation of vicinal hydroxyl groups via the action of a shortage of NaIO_4_ on the CPS, which was followed by a reductive amination of the resulting aldehydes in the presence of proteins (25). The products were isolated by Fractogel TSK-65 gel-permeation chromatography and characterized by electrophoresis in 12% polyacrylamide gel using the Laemmli method (26).

The oligosaccharides for conjugation by squaric acid chemistry were produced by cleavage of the K9 CPS with a recombinant depolymerase of a type K9-specific bacteriophage (24). They were fractionated by Fractogel TSK HW-40 (S) gel permeation chromatography and analyzed by 1D and 2D NMR spectroscopy as well as high-resolution electrospray ionization mass spectrometry. As a result, the structures of the oligosaccharides were established, and the linkage cleaved in the CPS by the depolymerase was determined.

**FIG 1:**
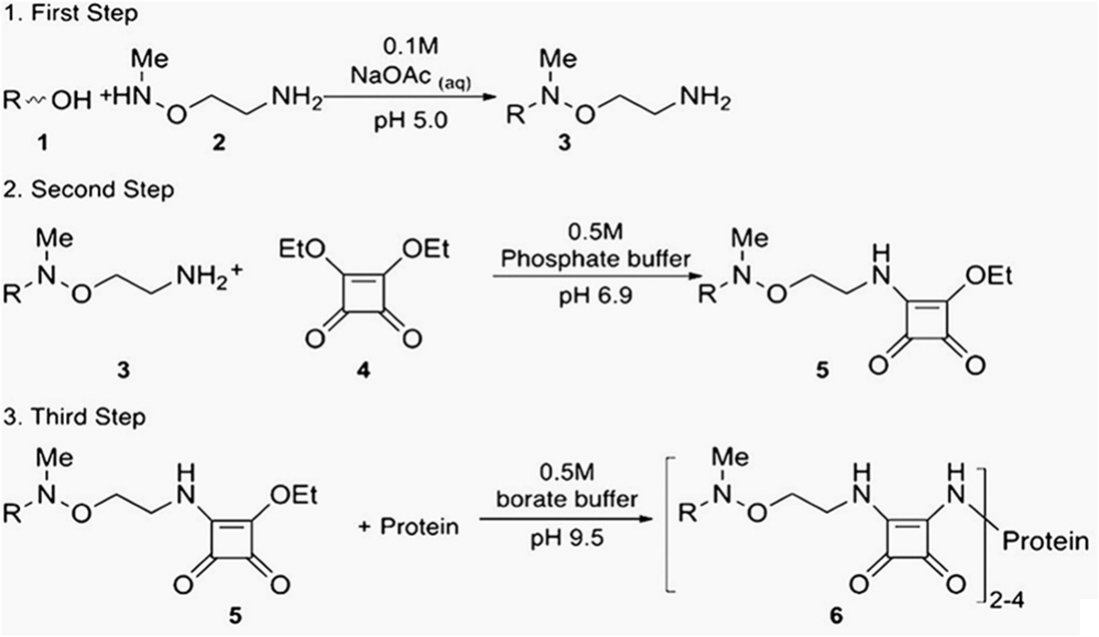
Synthesis of glycoconjugates by squaric acid chemistry.

The oligosaccharides that corresponded to two or three CPS monomers (K-units) were modified at the reducing end by 3-(methoxyamino)ethylamine in acetate buffer at pH 5 (27), as shown in Fig. 1. The resulting glycosides were converted into squaric acid derivatives by the action of diethylsquarate in phosphate buffer at pH 7.0; under these conditions, only one squarate ester group reacted (28). The products were conjugated with a protein (bovine serum albumin [BSA], chicken egg albumin [OVA] and snail hemocyanin [KLH]) in borate buffer at pH 9.5; under these conditions, another squarate ester group reacted. Intermediate products were isolated by Fractogel TSK HW-40 (S) gel chromatography, and the glycoconjugates were purified by dialysis against water. Analysis by laser-assisted desorption ionization mass spectrometry showed the presence of two to four CPS oligosaccharide fragments per glycoconjugate molecule.

### Analysis of the immune response in BALB/c mice after immunization with conjugates of K9 CPS fragments with proteins.

**FIG 2:**
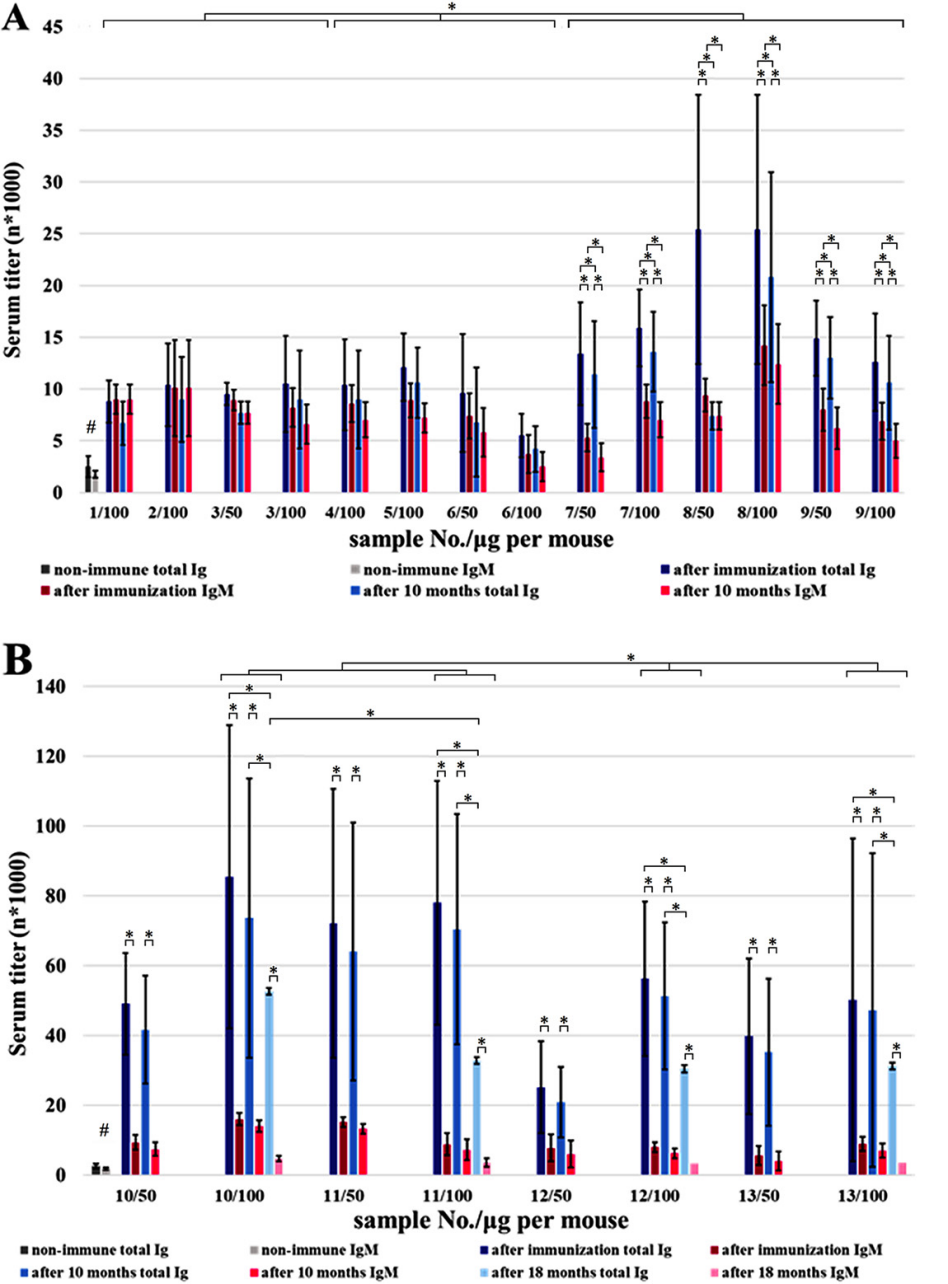
Titers of immune sera prepared by the immunization of BALB/c mice with type K9 capsular polysaccharide (CPS) (A) upon immunization with glycoconjugates obtained by periodate oxidation and (B) upon immunization with glycoconjugates obtained by squaric acid chemistry. After immunization, the sera were obtained on the seventh day after the last injection. Data are represented as means ± the standard errors of the means of 3 independent repeats (*n* = 10). The # symbol indicates a statistically significant difference (*P* < 0.05, Mann-Whitney) when the nonimmune sera were compared with each of the immune sera; * indicates a statistically significant difference (*P* < 0.05, Mann-Whitney).

Freund's adjuvant, as an adjuvant that has been proven to have high efficacy and is widely used in animal immunization, was chosen to start the study. The conjugates (Table 1) with Freund's adjuvant were used for the immunization of BALB/c mice (groups of 10 six to eight week females, about 20 g each). The immunization doses were 50 and 100 μg per animal. Blood was taken from the tail vein. The level of immune response was tested using an immunosorbent assay (EIA) by interaction of the blood sera of experimental animals with immobilized type K9 CPS. In each experimental group, the sera of all animals contained K9 CPS-reactive antibodies. Fig. 2 shows the relative content of the K9 CPS-reactive antibodies (titers) in sera from immunized BALB/c mice. The titers of total immunoglobulins and IgM, determined using an EIA by interaction with the immobilized K9 CPS, are shown for comparison. The titer of the immune serum was defined as a minimal dilution in which the binding of the K9 CPS was twice the background value.

**TABLE 1:** Glycoconjugates used for immunization

Periodate oxidation conjugation		
Sample no.	Fraction no. from TSK-65 column^*a*^	Carrier protein
1	6 to 7	Bovine serum albumin (BSA)
2	8 to 10	
3	11 to 13	
4	6 to 7	Chicken egg albumin (OVA)
5	8 to 10	
6	11 to 13	
7	6 to 7	Keyhole limpet hemocyanin (KLH)
8	8 to 10	
9	11 to 13	
**Conjugation by squaric acid chemistry**		
**Sample no.**	**No. of K-units**	**Carrier protein**
10	2	Bovine serum albumin (BSA)
11	3	
12	3	Chicken egg albumin (OVA)
13	3	Keyhole limpet hemocyanin (KLH)

a Corresponds to the average molecular weight of the glycoconjugates.

The content of total immunoglobulins induced by glycoconjugates prepared by periodate oxidation was either equal to or slightly higher than the content of IgM. No clear dose-dependent relationship was observed in this group upon immunization with glycoconjugates (Fig. 2A). Using KLH as the carrier protein increased the immunogenicity of the glycoconjugates 1.5 to 2-fold compared to those prepared based on OVA and BSA. At 10 months after the last immunization, K9 CPS-specific antibodies were still present in the sera of experimental animals, with their titers having decreased by 20%. In general, the level of K9 CPS-binding antibodies was lower when glycoconjugates prepared by periodate oxidation were used compared with those prepared by squaric acid chemistry (Fig. 2B).

Immunization with glycoconjugates obtained by squaric acid chemistry (samples no. 10 to 13, Fig. 2B) increased the titers of total immunoglobulins several-fold compared to samples no. 1 to 9 (Fig. 2A and B). A dependence on the amount of the preparation was observed. Sera from animals that were immunized with a 100 μg preparation showed higher titers compared to those immunized with 50 μg (Fig. 2B). The titers of total immunoglobulins were significantly higher than IgM titers, which showed a higher content of K9-binding IgG compared to IgM in sera and, hence, a higher level of immunological memory.

The levels of K9-binding immunoglobulins, both total and IgM, at 10 and 18 months after the last immunization are shown in Fig. 2B. At 10 months, the content of total immunoglobulins decreased by 17% to 11% in the groups immunized with 50 μg but by 13.8% to 6% in the groups immunized with 100 μg. Hence, the latter dose was more efficient for maintaining the level of immune response. At 18 months after the last immunization, when the animals were of old age, the levels of antibodies in sera their was rather high. The level of the immune response decreased by 38% when preparations no. 10 and 13 were used or by approximately twice that with no. 11 and 12. When BSA was used as carrier protein, the level of the immune response was approximately 25% higher than those obtained using OVA and KLH.

Glycoconjugate no. 10, which contains two monomer K-units, was a more effective immunogenic than was glycoconjugate no. 11, which contains three monomer K-units. A long-term preservation of the immune response level was observed for practically all glycoconjugates used.

### The immunological cross-reactivity of the immune sera produced by the immunization of BALB/c mice with conjugates of the K9 CPS fragments with carrier proteins prepared by squaric acid chemistry.

**FIG 3:**
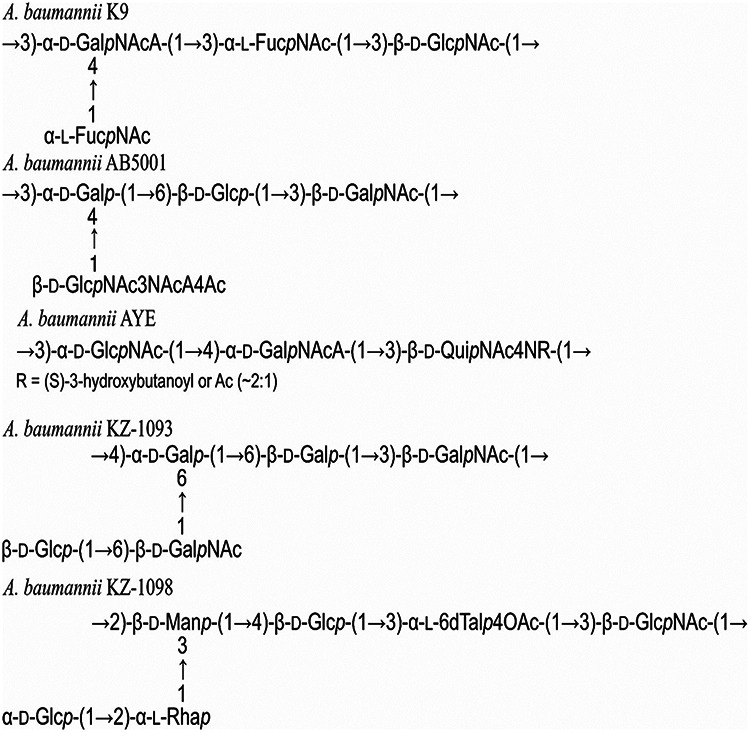
Chemical structures of A. baumannii K9, AB5001, AYE (29), KZ-1093 (30), and KZ-1098 (31) CPSs.

The immunological cross-reactivity of the immune sera obtained via immunization with glycoconjugates based on the K9 CPS fragments was studied by interaction with other A. baumannii CPSs, including AYE = MG1 (29), AB5001 (authors’ unpublished data), KZ-1093 (30), and KZ-1098 (31) (Fig. 3), as well as the deacylated forms of AB5001 и KZ-1098, compared to the K9 CPS. The AYE, К9, and АВ5001 CPSs are acidic, as they contain a uronic acid (GalNAcA or GlcNAc3NAcA). In АВ5001, a GlcNA3NAcA residue is present in the side chain; and in AYE and К9, a GalNAcA residue is present in the main chain. The СPSs of KZ-1093 and KZ-1098 are neutral.

**FIG 4:**
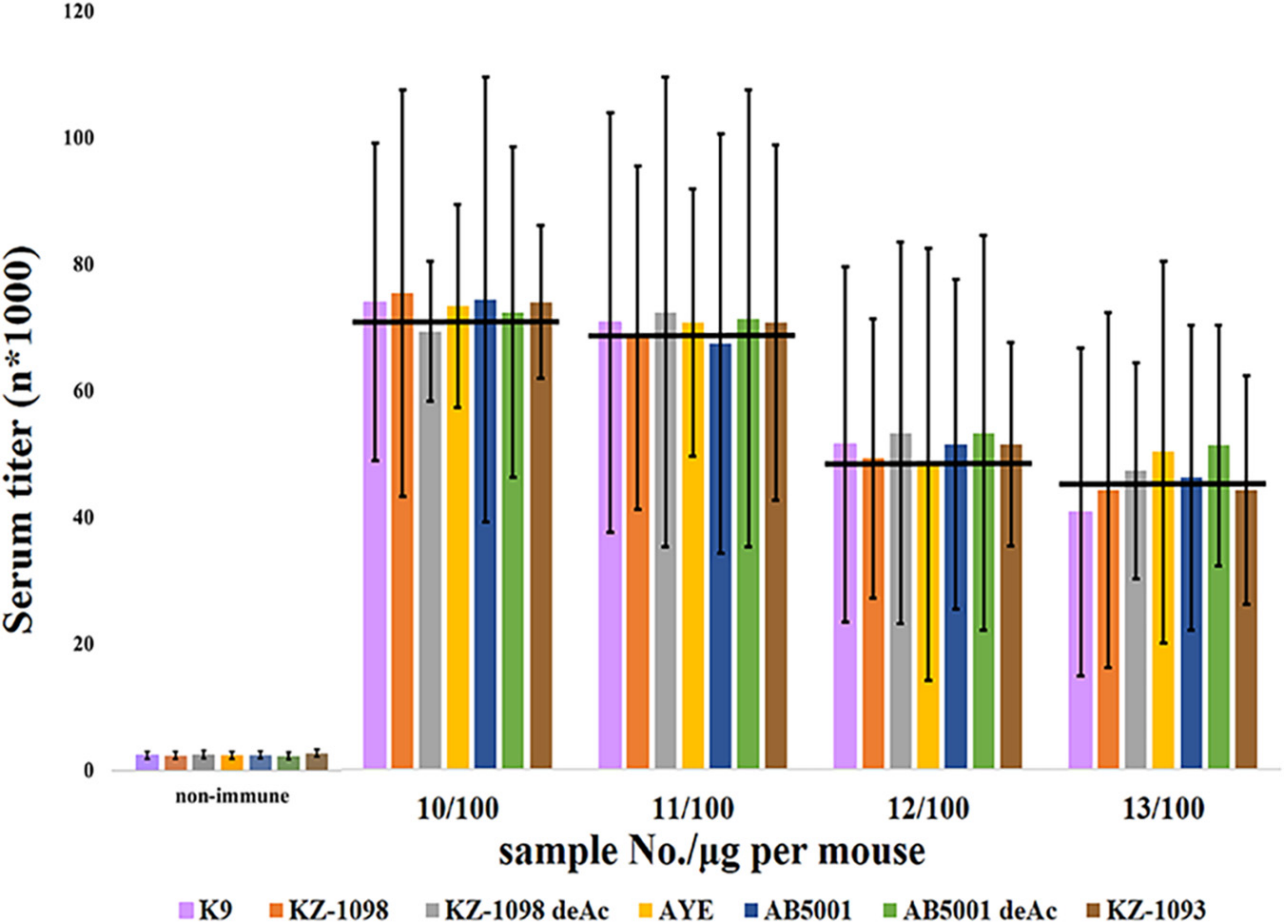
Titers of immune sera upon interaction with immobilized A. baumannii CPSs. The titers of immune sera were prepared by immunization of BALB/c mice with glycoconjugates (100 μg per mouse) obtained by squaric acid chemistry based on K9 CPS fragments. Data are represented as means ± the standard errors of the means of 3 independent repeats (*n* = 10). Medians are shown by horizontal lines. There is no statistically significant difference in serum (10/100, 11/100, 12/100, 13/100) titers for interactions with the listed CPSs (*P* < 0.05, Mann-Whitney).

No significant differences were observed in the binding of the sera obtained at 10 months after the last immunization with preparations no. 10 13 (Fig. 4).

**FIG 5:**
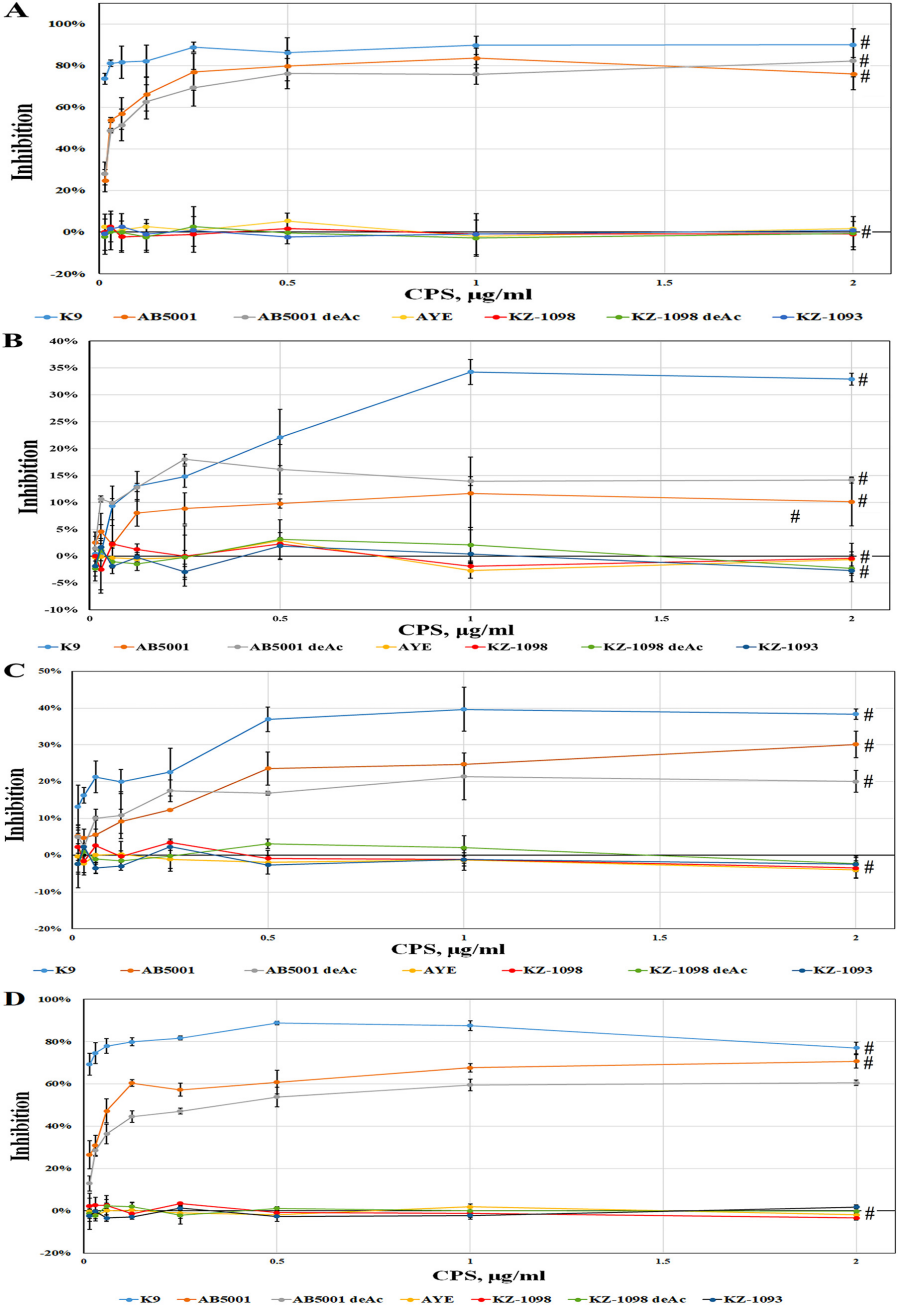
Inhibition of the interaction of immune sera 10/100 (А), 11/100 (В), 12/100 (С), 13/100 (D) with K9 CPS by various CPSs. Data are represented as means ± the standard errors of the means of 5 independent repeats. The # symbol on the charts indicates a statistically significant difference (*P* < 0.05, Mann-Whitney) in comparison with the theoretical maximum inhibition (100%) corresponding to the background values of the reactions.

Inhibition was applied to confirm the specificity of the interactions of the immune sera with the immobilized CPSs. In assay 1, the binding of the immune sera with the immobilized K9 CPS was inhibited by each CPS from the present set (Fig. 5). The AYE, KZ-1093, and KZ-1098 CPSs, as well as the deacylated KZ-1098 CPS, did not inhibit the interactions of the 10/100 (Fig. 5A), 11/100 (Fig. 5B), 12/100 (Fig. 5C), and 13/100 (Fig. 5D) immune sera with the immobilized K9 CPS. The CPSs of K9 and AB5001, as well as the deacylated form of the AB5001 CPS, inhibited this interaction with different degrees of effectiveness.

The K9 СPS, fragments of which were used for immunization, inhibited the interaction of 10/100 serum (Fig. 5A) most effectively. At a concentration of 2 μg · mL^−1^ of the inhibitor, the binding of the 10/100 serum decreased by 90%, while the 13/100 (Fig. 5D), 12/100 (Fig. 5C), and 11/100 (Fig. 5B) sera decreased by 77, 45, and 33%, respectively. The interaction of the K9 CPS was also inhibited by the AB5001 CPS (by 76, 71, 30, and 10%), and to a lower degree, by the deacylated AB5001 CPS (60, 60, 20, and 14%, respectively). The high level of inhibition of the binding of the sera with the K9 CPS by the AB5001 CPS suggests a similarity between their epitope structures.

**FIG 6:**
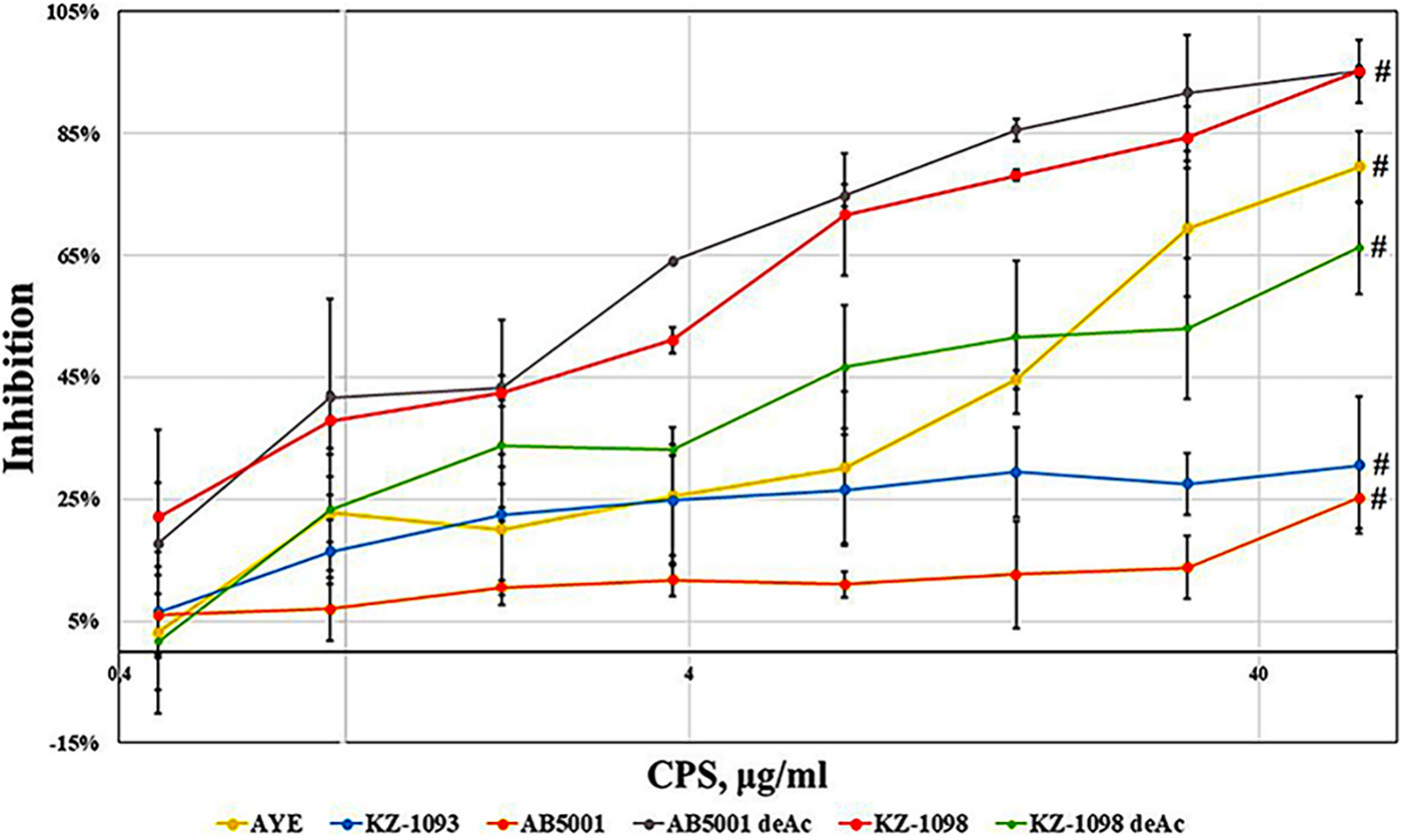
Inhibition of the interaction of 10/100 serum with each of the six immobilized CPSs by each of these CPSs. Data are the means ± the standard errors of the means of 5 independent repeats. The # symbol on the charts indicates a statistically significant difference (*P* < 0.05, Mann-Whitney) in comparison with the theoretical maximum inhibition (100%) corresponding to the background values of the reactions.

In assay 2, the inhibition of the binding of sera 10/100, 11/100, 12/100, and 13/100 (Table 1) (100 μg per mouse) with each sorbet polysaccharide from the above list was performed by the same polysaccharide to which the serum bound in this experiment, that is, sorbed on plastic. The lowest specificity was demonstrated by the 11/100 serum. Its interaction with the immobilized AB5001 CPS was inhibited by the AB5001 CPS, either deacylated or not, by 40%, and the interaction with the other antigens was inhibited by 15 to 25% only (Fig. 6) at an inhibitor concentration of 60 μg · mL^−1^.

Similar results were obtained with the 10/100, 12/100, and 13/100 sera. The AYE CPS inhibited the interaction with the immobilized AYE CPS, on average, by 75 to 80%; the AB5001 CPS by 25 to 40%; the deacylated AB5001 CPS by 70 to 95%; the KZ-1093 CPS by 20 to 40%; the KZ-1098 CPS by 70 to 95%; and its deacylated form by 55 to 70% (at an inhibitor concentration of 60 μg · mL^−1^). Fig. 6 shows the titration curves for the 10/100 serum when the same CPS was immobilized for the EIA and used for inhibition.

The obtained results showed different levels of specificity in respect to both of the CPS sets used and each serum type, depending on the glycoconjugate taken for immunization (Table 1). These data could be accounted for by a complex structure of the polysaccharides which presumably contain various epitopes. Antibodies to these epitopes can be produced in experimental animals and therefore may occur in polyclonal serum.

### Determination of the type of immune response in BALB/c after immunization with conjugates prepared by squaric acid chemistry.

The contents of subclasses IgG2a and IgG2b in serum are linked to the Th1 response and that of IgG1 to the Th2 response. Immunization may change not only the ratios of antigen-specific IgG subclasses which show a link to Th1 or Th2 but also the general ratios of the IgG subclasses in blood serum (32–34).

The immune response was characterized using an EIA, as both the total ratios of subclasses IgG2a/IgG1 and IgG2b/IgG immunoglobulins in blood serum and the ratio of subclasses of type K9 CPS specific antibodies in sera obtained 10 months after immunization with the preparations based on squaric acid chemistry.

**FIG 7:**
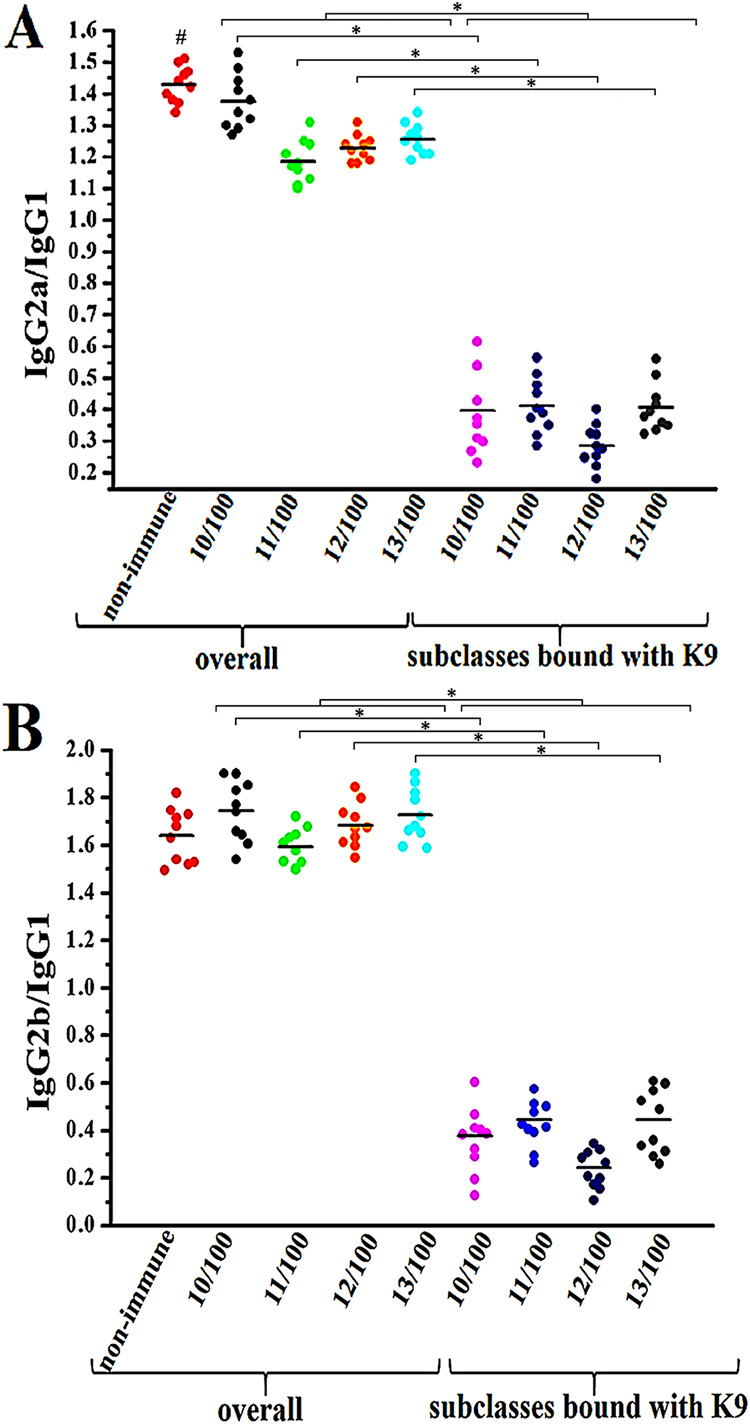
Ratios of IgG subclasses in the sera of BALB/c mice immunized with preparations based on K9 CPS fragments obtained by squaric acid chemistry (the number and dose of a preparation in μg per animal are shown). Panels А and B show the IgG2a/IgG1 and IgG2b/IgG1 ratios, respectively, in the sera of experimental animals. A serum sample from each experimental animal was analyzed in triplicate. The figure indicates the average values. Medians are shown by horizontal lines (*n* = 10). The # symbol shows a comparison of the overall ratio of nonimmune and immune sera in panel A (*P* < 0.05, Mann-Whitney); * indicates a statistically significant difference between data groups marked with brackets (*P* < 0.05, Mann-Whitney).

Before immunization in normal mouse sera, the average IgG2a/IgG1 ratio was 1.43, and after immunization in immune sera, this ratio decreased by an average of 11% (Fig. 7A). Determining the ratio of IgG subclasses interacting with K9 revealed a significant decrease in the IgG2a/IgG1 ratio. The mean values ranged from 0.29 for a serum obtained from an animal immunized with preparation no. 12 to 0.41 for a serum obtained from an animal immunized with preparation no. 11. Figure 7A shows that the ratios of antibodies that bind to K9 (IgG2a/IgG1) overlap to a large extent.

The average IgG2b/IgG1 ratio in normal mouse sera was 1.64 before immunization and almost the same in immune sera after immunization (Fig. 7B). Determination of the K9-reactive IgG subclass ratios showed a significant decrease of the IgG2b/IgG1 ratio. The average value varied from 0.24 for a serum from an animal immunized with preparation no. 12 to 0.45 for the 13/100 serum. Similar to the K9-reactive IgG2a/IgG1 ratio, the IgG2b/IgG1 ratios overlapped at some experimental points.

These data correspond to the finding that the dominant subclass IgG antibodies’ response to a polysaccharide switches to the IgG1 response when the polysaccharide is conjugated to a protein in both mice and people (35).

### Analysis of lymphokine production after immunization with glycoconjugate.

Data on the ratio of immunoglobulin subclasses in immune sera after the immunization of BALB/c mice with different glycoconjugates indicate the presumed development of an immune response by the Th2 type. The immunization scheme provided for the use of a full Freund’s adjuvant, the components of which modulate immune responses. To study the features of the immune response to glycoconjugates, additional experiments, in which there was no additional stimulation of the immune response by adjuvants, were carried out to immunize the BALB/c and ICR mice. The ICR mice, unlike the BALB/c animals, were outbred and had no predisposition for this type of adaptive immune response.

**FIG 8:**
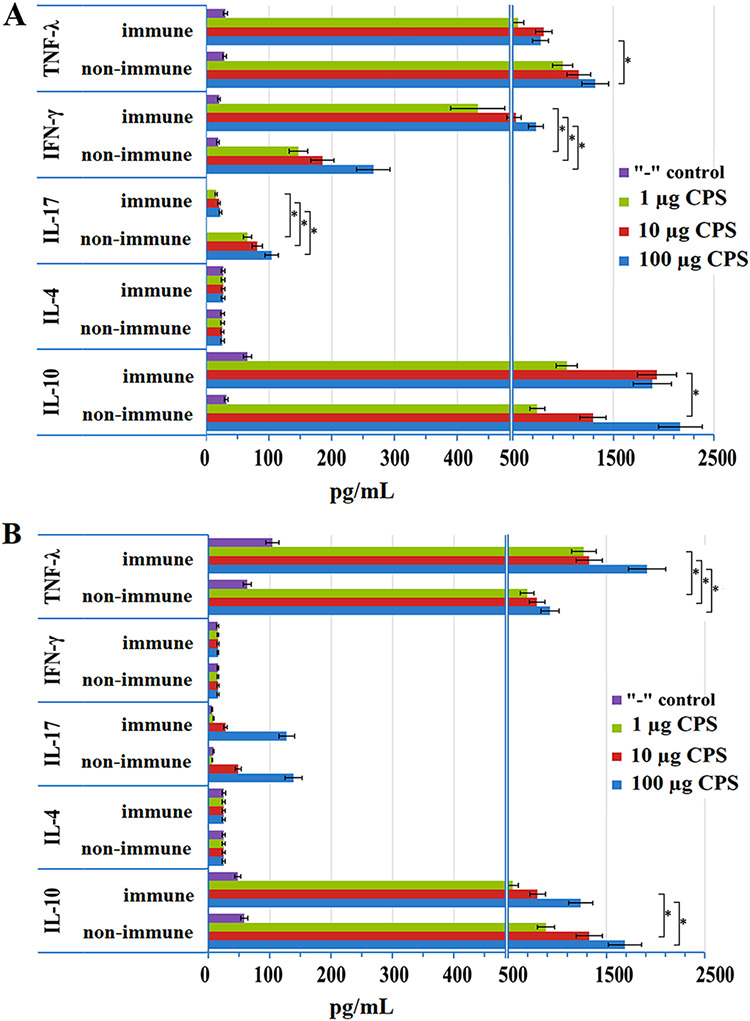
Analysis of lymphokine production after the *in vitro* K9 CPS stimulation of splenocytes isolated from the spleens of mice immunized with glycoconjugate no. 10 (Table 1) nonimmunized ICR (A; *n *= 10 mice/group), and BALB/c (B; *n *= 10 mice/group). Data are represented as means ± the standard errors of the means of 3 independent repeats. * indicates a statistically significant difference (*P* < 0.05, Mann-Whitney).

Splenocytes were isolated from immunized and nonimmunized animals that were cultured in a medium with different contents of K9 CPS, which was the source of the carbohydrate fragment in the synthesis of glycoconjugate. After that, the contents of IL-4, IL-10, IL-17A, γ-IFN, and TNF-α were analyzed (Fig. 8). The analysis revealed a significant difference in the profile of secreted cytokines. It turned out that in the BALB/c mice there was no antigen-induced expression of γ-IFN, while the ICR mice featured a pronounced antigen concentration-dependent expression of this lymphokine. Moreover, the production of γ-IFN was detected both in immune splenocytes and in splenocytes obtained from nonimmune animals, albeit in smaller amounts. The expression of γ-IFN upon the *in vitro* K9 CPS stimulation of nonimmune splenocytes reflects the response of the innate immune system. When immune splenocytes were stimulated, the production of γ-IFN was significantly higher, which can apparently be explained by the work of the adaptive immunity system, namely, the T-lymphocytes. The production of TNF-α and IL-10 was also observed; however, for these lymphokines, there were practically no differences between the immune and the nonimmune splenocytes. Neither the BALB/c mice nor the ICR mice expressed IL-4 in response to polysaccharide stimulation.

Taken together, these data indicate that the adaptive immune response develops by the Th1 type at this stage. Also, the stimulation of splenocytes *in vitro* causes a dose-dependent activation of IL-17A expression in both BALB/c and ICR mice. At the same time, the levels of expression of IL-17A by CPS-stimulated splenocytes from nonimmunized mice was higher than those of splenocytes from animals that received two injections of the glycoconjugate. These data indicate the activation of the immune response mechanisms at the initial stage and by the Th17 type.

### Opsonisation activity of immune sera.

**FIG 9:**
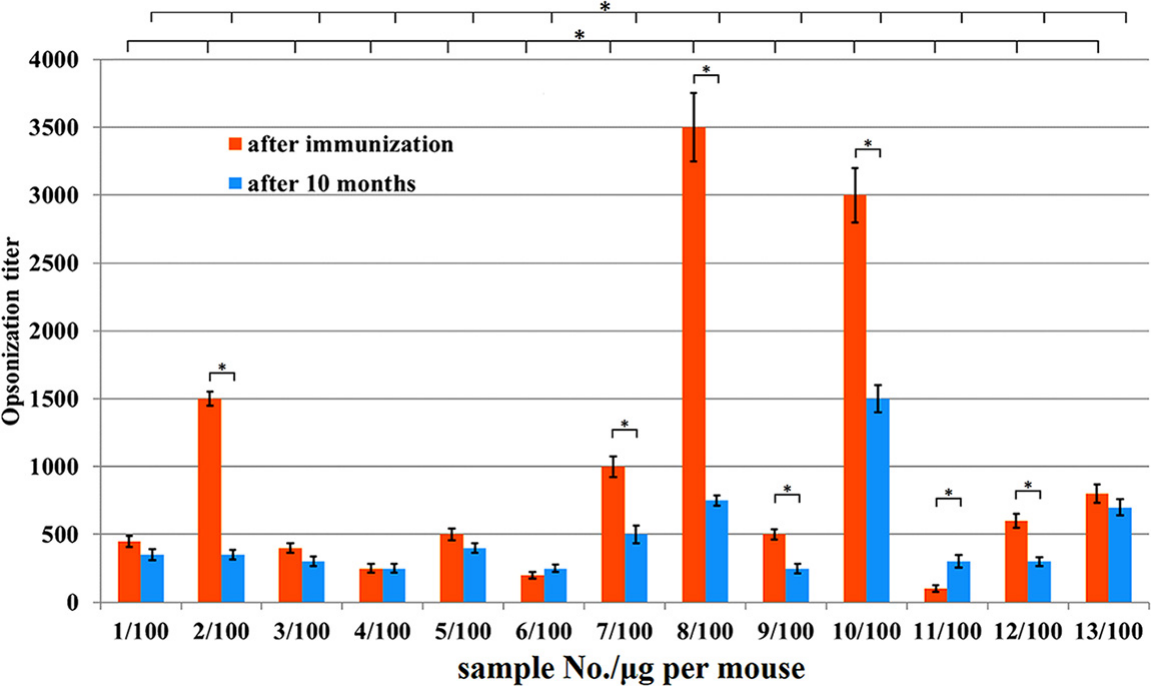
Opsonization titers of A. baumannii K9 bacteria by immune serum. Data are represented as means ± the standard errors of the means of 3 independent repeats. * indicates a statistically significant difference (*P* < 0.05, Mann-Whitney) that was only observed for the data sets marked by brackets.

The serum opsonization ability of immunized mice toward A. baumannii was estimated by a decrease in the number of colonies, that is, the bacteria that were absorbed by macrophages (cell line J774). The number of colonies at experimental points after incubation with their sera studied was compared with the number of colonies after incubation with the nonimmune serum in the same dilution (negative-control). The dilution of the serum studied, which caused a 50% difference with the negative-control, was regarded as the opsonization ability. The opsonization ability of the immune sera toward A. baumannii strain K9 is shown in Fig. 9. The opsonization titers of the immune sera, obtained on the seventh day after the last injection and at 10 months after immunization, were compared. The highest opsonization titers were observed for the 10/100 serum (squaric acid chemistry) and the 8/100 serum (periodate oxidation). The opsonization titers of the sera that were studied were preserved during a 10-month observation period.

**FIG 10:**
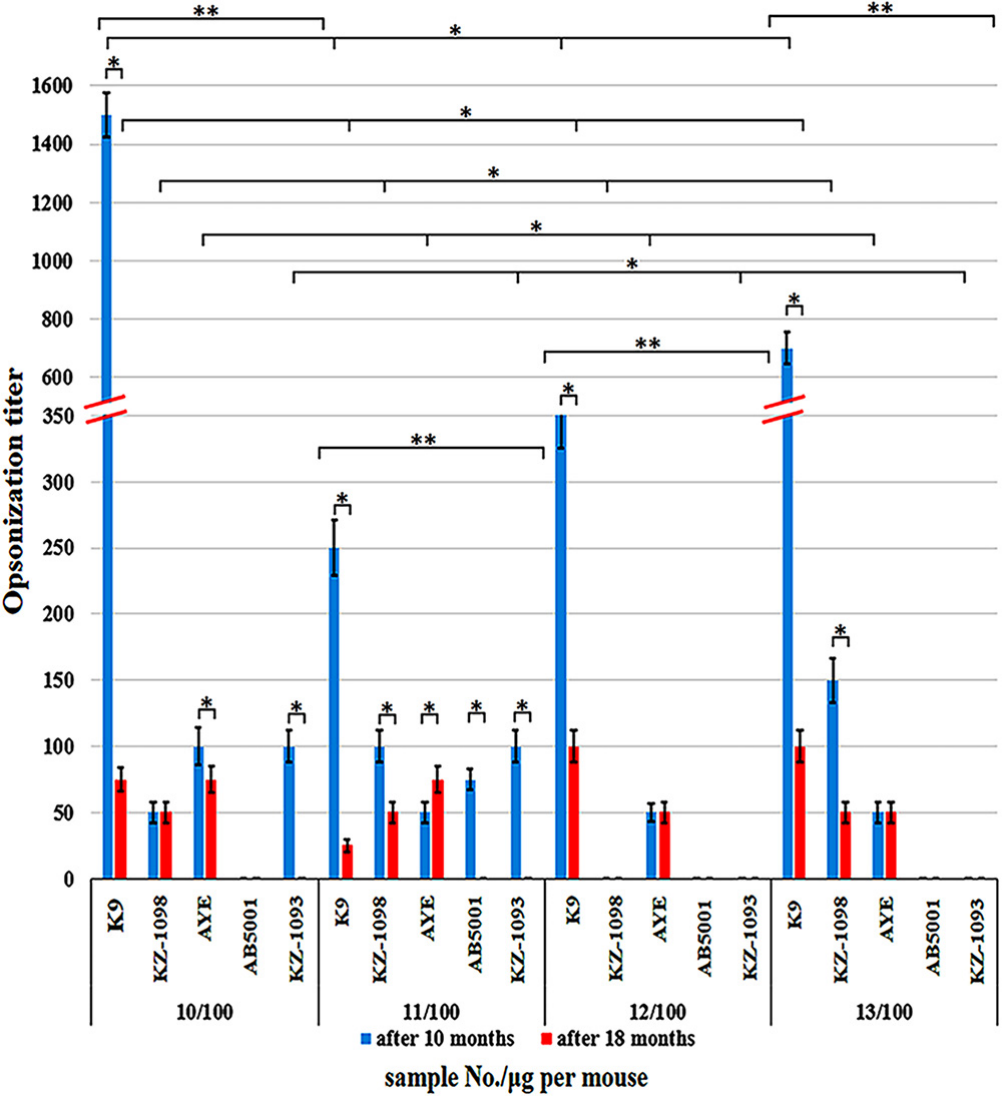
Opsonization titers of A. baumannii K9, KZ-1098, AYE, AB5001, and KZ-1093 bacteria by immune serum. Data are represented as means ± the standard errors of the means of 3 independent repeats. * indicates a statistically significant difference (*P* < 0.05, Mann-Whitney) when comparing the ability of each of the sera to opsonize a particular strain and the ability to opsonize a particular bacterial strain with different sera. ** indicates a statistically significant difference (*P* < 0.05, Student’s *t* test) when comparing the results fn the ability of a particular serum to opsonize different strains of A. baumannii.

The opsonization activity of the serum obtained after immunization with conjugates prepared by squaric acid chemistry toward A. baumannii strains K9, KZ-1098, AYE, AB5001, and KZ-1093 is shown in Fig. 10. At 10 and 18 months after immunization, none of the immune sera studied opsonized strain AB5001, except for the 11/100 serum, which lost this ability after 18 months. Strain KZ-1098 was not opsonized by the 12/100 serum only. At 10 months, strain KZ-1093 was not opsonized by the 12/100 serum or the13/100 serum. At 18 months, all of the sera studied lost their abilities to opsonize this strain. All of the sera studied effectively opsonized the homologous strain K9.

At 10 months after immunization, all sera demonstrated a relatively high opsonization level with respect to the “native” strain K9, which was significantly higher than those with respect to the cross-reactive strains, and demonstrated preserved opsonization abilities, although they were lower after 18 months. Apart from IgM, the antibodies of the IgG2a and IgG2b subclasses were known to possess the highest opsonization abilities. These antibodies interacted with single activating receptor, FcγRIII, located on both myeloid and NK cells, and they contributed the most to combating against the infection by antibody-dependent cell cytotoxicity (ADCC). This represents a mechanism by which the effectory cells of the immune system lyse target cells whose surface membrane antigens are linked to specific antibodies (36, 37).

### Survival against a challenge with A. baumannii.

**FIG 11:**
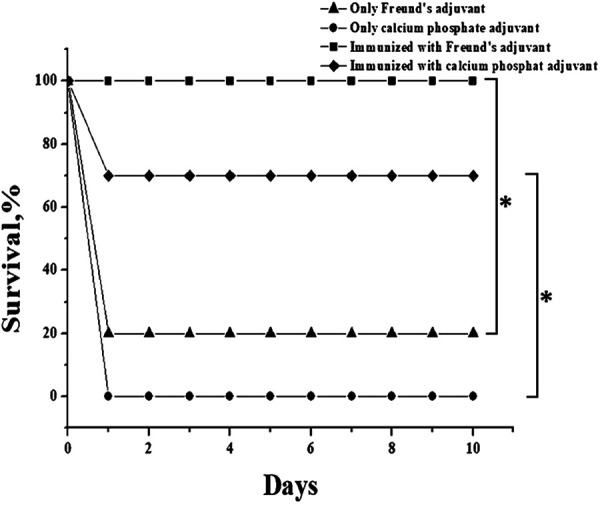
Survival of mice immunized with preparation no. 12 and control mice after infection with a lethal dose (10^8^ CFU/mouse) of A. baumannii K9 (*n *= 10 mice/group). * displays a statistically significant difference between the adjuvant’s control group and the data groups marked with brackets (*P* < 0.05, nonparametric log-rank test).

Preparation no. 12 was selected to be used to check the protective ability of the glycoconjugates against infection. This represents a midlevel preparation in terms of the immune response level and the opsonization ability among the conjugates obtained by squaric acid chemistry. For this purpose, immunized mice were infected by bacteria at a lethal dose of 10^8^ colony forming units (CFU)/mouse at 2 weeks after the last immunization. As a result, the mice immunized by the glycoconjugate no. 12 (Table 1) were fully protected from infection and were well during the entire observation period. At the same time, 80% of the control animals, which received only injections of Freund's adjuvant, died on the first day after infection (Fig. 11).

Experiments on the survival of animals after a challenge with A. baumannii were also carried out by using calcium phosphate as an adjuvant, representing a compound that is non-toxic to animals and to humans and is used for vaccination (38). The results are shown in Fig. 11. After the challenge, 70% of the immunized animals survived against the background of 100% mortality in the group that received only calcium phosphate. The data obtained indicate a high protective ability of the glycoconjugate obtained by square acid chemistry against an A. baumannii infection.

**FIG 12:**
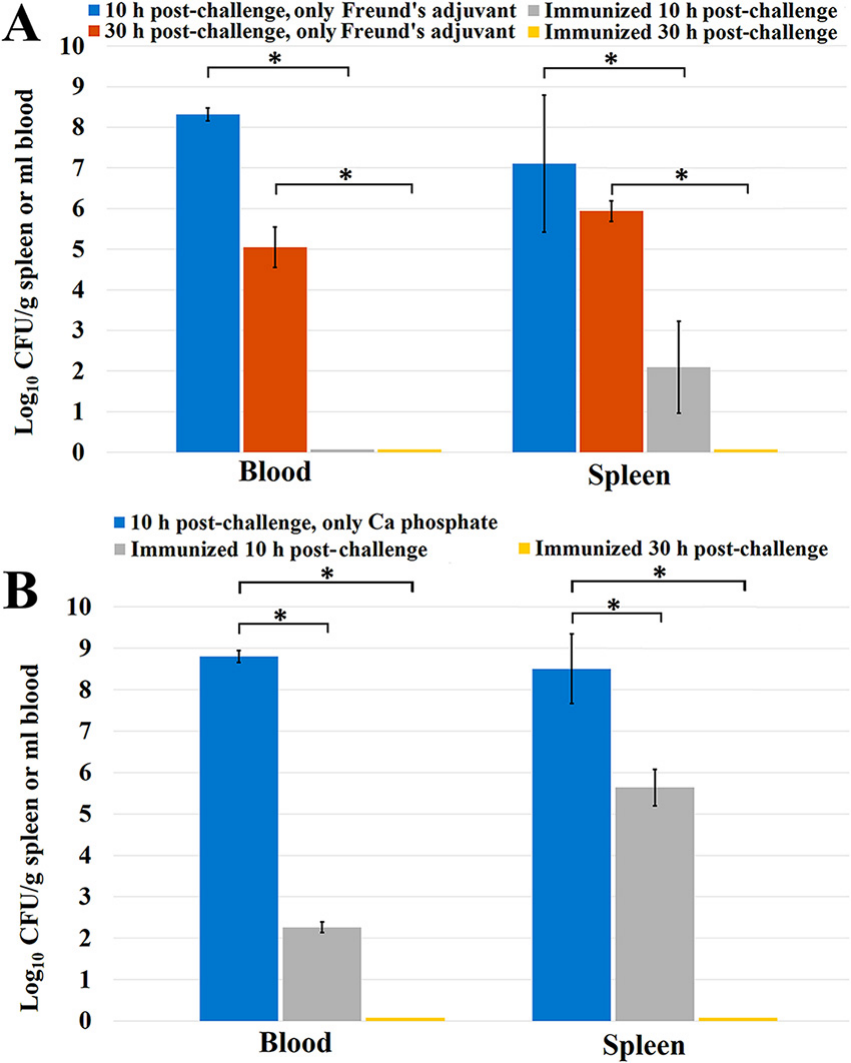
Bacterial load was evaluated in the spleen and blood of immunized and control mice at 10 and 30 h postinfection (*n *= 5 mice/group). BALB/c mice were immunized with preparation no. 12 at a dose of 100 μg/mouse (A) Using Freund's adjuvant. In the case of the control group, only 2 mice (*n *= 2) were used at the 30 h experimental point. (B) Using calcium phosphate. Data are represented as means ± the standard errors of the means of 3 independent repeats. * indicates a statistically significant difference for the CFU (*P* < 0.05, Mann–Whitney) between the data groups marked with brackets.

To estimate the influence of immunization on the bacterial burden at 10 h after infection, when the control mice were in poor condition and and near death, 5 animals from each of the immunized and control groups were killed. Their blood and spleens were analyzed to determine the bacterial loading (Fig. 12A). It was found that at 10 h after infection with a lethal dose of the inoculant, immunization decreased the content of the bacteria in the spleen by 5 to 6 orders of magnitude and completely freed the blood from bacteria. No bacteria were found in the blood and spleens at 30 h after infection, thus indicating the complete removal of the infectious agent. The determination of the bacterial load in a small number of the surviving mice that received only injections of Freund's adjuvant showed its reduction to a much lesser extent than that observed in the immunized mice.

In the case of using calcium phosphate as an adjuvant (Fig. 12B), the content of bacteria in the spleen at 12 h after infection decreased to a lesser extent than that observed when using Freund's adjuvant, representing a decrease of about 3 orders of magnitude, with a decrease of about 5 orders of magnitude being observed in the blood. The determination of the bacterial load in the blood and spleen 30 h after infection did not reveal the presence of bacteria in the test samples, indicating that the infectious agent was eliminated in both the mice immunized with Freund's adjuvant and those treated with calcium phosphate.

## DISCUSSION

Infections caused by A. baumannii are of concern, owing to their increasing prevalence and antibiotic resistance. They are common in high-risk groups, including patients in intensive care units and those on a ventilator. Therefore, the development of new vaccines has become an urgent issue (39).

There is successful world experience with which to apply glycoconjugate vaccines. The first polysaccharide-protein conjugate vaccine against Haemophilus influenzae serotype b (Hib) was licensed in the USA in 1987, and it not only decreased morbidity but also eliminated newborn carriers and, as a result, protected the entire population via collective immunity (40). Conjugated vaccines based on Streptococcus pneumoniae capsular polysaccharides have been under study worldwide in recent decades. In spite of the problems due to pathogen variability, the application of these vaccines prevents carriage, provides collective immunity, and decreases the morbidity of pneumococcal infections in newborns and adults (41).

A number of vaccine preparations have been reported as being able to provide protection against several strains in animal models, and computer programs have been developed to identify novel potential vaccine candidates against A. baumannii (42).

The carbohydrate components of the existing conjugate vaccines against A. baumannii are pseudaminic acid for the protection of mice against infections by pseudaminic acid-producing strains (43) and poly-N-acetyl-β-(1-6)-glucosamine (PNAG), which is expressed by a wide range of bacterial pathogens (44). Outer membrane vesicles decorated with PNAG possess a high protective potential against bacterial pathogens displaying PNAG on their cell surfaces (45). Another conjugate vaccine against A. baumannii was developed by introducing an O-linked glycosylation system into the host strain via a biosynthetic method (termed protein glycan coupling technology) (46).

The present study is aimed at the elaboration of approaches by which to construct glycoconjugate vaccines against infections caused by A. baumannii, since there is successful world experience in the use of such vaccines (e.g., Haemophilus influenzae serotype b, Streptococcus pneumoniae). The polysaccharide-protein conjugate approach has not been previously used for A. baumannii (21).

13 glycoconjugates that contained fragments of various lengths of the A. baumannii type K9 CPS, contained various carrier proteins, and were obtained via two methods of chemical conjugation (Table 1) were compared by their immune response levels and characterized by both their relative contents of immobilized type K9 CPS-reactive specific antibodies and their abilities to opsonize A. baumannii K9. Using squaric-acid glycoconjugates for immunization induced more IgG than IgM, which demonstrated the involvement of cells responsible for the adaptive immune response and, hence, the immunological memory. As demonstrated by the analysis of the immune sera obtained at 10 and 18 months after the immunization of the mice, the animals preserved the titers of the specific immunoglobulins for practically their entire lifetimes.

The following was the optimal composition of the glycoconjugate synthesized by squaric acid chemistry: a type K9 CPS fragment composed of two CPS monomer units with BSA as the carrier protein.

The immune sera demonstrated a high specificity toward the “native” type K9 CPS. In competitive EIA, the interaction of the 10/100 serum with the immobilized type K9 CPS was practically completely inhibited by the free K9 CPS. Possible limitations of the immune-cross-reactivity could be due to differences in the accessibility of carbohydrate antigenic determinants in the dissolved and immobilized CPSs for antibodies.

The immune sera effectively opsonized the type K9 strains and, to a lesser extent, the immuno-cross-reactive strains. The opsonization ability of the sera decreased with time but remained sufficiently effective until the mice reached old age. When using Freund's adjuvant with BALB/c mice, the immune response was developed predominantly by the Th2 type, and, hence, subclass IgG1 antibodies, which are not involved in opsonization and are instead responsible for ADCC, dominated among the specifically K9-reactive antibodies. Therefore, in spite of the predominant content of IgG1 in the sera, the antibodies of other subclasses and IgM ensured a high level of opsonization activity.

The Th1/Th2 balance of adaptive immunity plays a crucial role in the outcomes of many diseases (47). It is known that in BALB/c mice, there is a genetically determined predisposition to the formation of adaptive immune responses of the Th2 type (48). We compared the secreted lymphokine profiles of BALB/c and ICR mice after immunization with glycoconjugate and without the additional stimulation of the immune response by adjuvants.

It turned out that the ICR mice, unlike the BALB/c mice, experienced activation of γ-IFN production, which is an important factor of innate and adaptive immunity. It is known that γ-IFN can be produced by CD4^+^ Th1 lymphocytes. With the help of γ-IFN, Th1-lymphocytes activate macrophages, and this leads to an active inflammatory reaction aimed at eliminating pathogens. The production of IL-10, IL-17A, and TNF-α, but not IL-4, was also activated to various degrees. TNF-α is a proinflammatory cytokine with a wide range of activities. In combination with γ-IFN, it significantly enhances the bactericidal potential of phagocytes. IL-10, in contrast, belongs to the group of anti-inflammatory cytokines. IL-10 has previously been shown to play an important role in determining the outcome of A. baumannii infections by protecting lung tissues from fatal damage due to inflammatory responses (49). That is, it is important to maintain a balance of inflammatory and anti-inflammatory mediators of immune response. Therefore, the observed picture of the profile of lymphokines after immunization with glycoconjugate and the *in vitro* stimulation with a polysaccharide are encouraging in terms of their potential use in the development of a vaccine against A. baumannii.

The absence of IL-4 expression induced by K9 CPS and the glycoconjugate fragment of this polysaccharide are indicative of the development of a Th1-type adaptive immune response. Prior work has demonstrated the role of IL-17 in the formation of adaptive immunity against A. baumannii; Th17 cells are able to attract and activate neutrophils, which contribute to protection against extracellular pathogens. IL-17A plays a key role in preventing both infection progression and associated infectious complications (50). However, the weak response of IL-17A to CPS stimulation of immune splenocytes indicates that the Th17 response is somehow blocked. It is known that lymphokines produced by Th1 and Th2 cells can have an antagonistic effect on the Th17 population (51). Also, it is likely that in the initial stage of the immune response, the innate immune system is activated, and adaptive responses are modulated by the type of Th1 and Th17. However, as the population of antigen-specific Th1 cells increases, the lymphokines produced by them inhibit the functioning of the Th17 cells. In this way, the unwanted damaging effects of Th17 cell activity are prevented.

The infection of immunized animals with A. baumannii K9 demonstrated a high protective ability of the glycoconjugate toward the infection. All of the immunized mice survived after the administration of the lethal dose of bacteria. In the case of using calcium phosphate as an adjuvant, the survival rate was 80%. No bacteria were detected in the blood and spleens of immunized animals at 30 h after the challenge with a lethal dose.

## MATERIALS AND METHODS

### Periodate oxidation.

Solution А: 8 mM NaIO_4_ (1.68 g · L^−1^) in deionized water was prepared on the day of application and protected from direct light. Solution B: 0.1 М NaHCO_3_ (8.4 g · L^−1^).

1 mL of solution A was added to a solution of 20 mg CPS in 1 mL of solution B. After 2 h at 20°С in the dark, a solution of 20 mg protein in 1 mL of 0.1 М NaHCO_3_ (pH 9.2) was added. The mixture was carefully mixed in a column with Sephadex G-25 and was incubated for 3 h at 20°С. After elution from the column, a 1/20 volume of a freshly prepared solution of NaBH_4_ (5 mg · mL^−1^ in 0.1 М NaOH) was added. After 30 min, a 3/20 volume of the same solution was added. After 1 h, the solution was applied to a Fractogel TSK-65 column (1.6 × 100 cm), and the product was eluted with a 1% solution of AcOH in water.

### Synthesis of a glycoconjugate with an intact oligosaccharide.

Oligosaccharide (6.7 μmol) was dissolved in a 1.47 М solution of 3-(methoxyamino)ethylamine in 0.1 M acetate buffer (100 μL, рН 5.2) and treated in a thermostat at 37°С for 48 h. The solution was applied to a Fractogel TSK HW-40 (S) column (1.6 × 80 cm), and the product was obtained with a yield of 90 to 95% by elution with a 1% solution of AcOH in water. The mass spectrum of the oligosaccharide with K-2 units showed a peak for an [M−H]-ion at *m/z* 1,678.6804 against the calculated value of *m/z* 1678.6892 (C_67_H_110_N_10_O_39_) and a peak for an [M−H]^−2^ ion at *m/z* 838.8449. The mass spectrum of the oligosaccharide with K-3 units showed a peak for an [M+H_2_O+HCl−H]^−2^ ion at *m/z* 1,262.8476 against the calculated value of *m/z* 2,474.0073 (C_67_H_110_N_10_O_39_).

The oligosaccharide with an amino ethyl spacer (6.7 μmol) was dissolved in phosphate buffer (1 mL, pH 7.0), and then diethylsquarate (34 μL, 39.2 mg, 0.23 μmol) was added. After 16 h at 20°С and centrifugation, the solution was applied to a Fractogel TSK HW-40 (S) column (1.6 × 80 cm), and the product (with a yield of 90 to 95%) was obtained by elution with a 1% solution of AcOH in water. The mass spectrum of the oligosaccharide with K-2 units showed a peak for an [M−H]^−2^ ion at *m/z* 900.3454 against the calculated value of *m/z* 1,802.7092 (C_73_H_114_N_10_O_42_). The mass spectrum of the oligosaccharide with K-3 units showed a peak for an [M−H]^−2^ ion at *m/z* 1,298.0058 against the calculated value of *m/z* 2597.0161 (C_105_H_164_N_14_O_61_).

The glycoside obtained (1.79 μM) (Table 2) was added to a solution of protein (0.388 μM) (Table 2) in a 0.5 M borate buffer (670 μL, pH 9.0). After 3 days, the solution was diluted with water and dialyzed to give the expected glycoconjugate.

**TABLE 2:** Amounts of oligosaccharides and protein taken for the synthesis of the glycoconjugates used for immunization

Sample no.	K-unit	Protein	K-unit: protein ratio
10	2 (3.6 mg, 1.2 nm)	BSA (22 mg, 0.26 nm)	~ from 2 to 4 (4 to 8% of oligosaccharide content)
11	3 (5.0 mg, 1.9 nm)	BSA (20.3 mg, 0.24 nm)	~ from 2 to 4 (4 to 8% of oligosaccharide content)
12	3 (4.8 mg, 1.8 nm)	OVA (13.4 mg, 0.29 nm)	~ from 2 to 4 (4 to 8% of oligosaccharide content)
13	3 (4.4 mg, 1.5 nm)	KLH (16.0 mg, 0.18 nm)	~ from 2 to 4 (4 to 8% of oligosaccharide content)

### Mass spectrometry.

High-resolution electrospray ionization mass spectrometry was performed in the negative ion mode using a micrOTOF II instrument (Bruker, Germany). Oligosaccharide samples (~50 ng · L^−1^) were dissolved in a 1:1 (vol/vol) water-acetonitrile mixture and injected with a syringe at a flow rate of 3 μL · min^−1^. The capillary entrance voltage was set at 3,200 V, and the interface temperature was 180°C. Nitrogen was used as a drying gas. The mass range was from *m/z* 50 to *m/z* 3,500. Internal calibration was done with the ESI Calibrant Solution (Agilent Technologies, USA).

MALDI-TOF mass spectra were registered on a Bruker Ultraflex II mass spectrometer that was equipped with the panoramic delay of ion extraction electronics with the registration of positive ions in a linear mode using an accelerating voltage of 20 kV. 2,5-dihydroxybenzoic acid was used as a matrix.

### Glycoconjugate isolation.

Gel permeation chromatography was performed on an XK 16/100 column (1,000 × 16 mm, gel layer, 800 mm) (GE Healthcare) filled with Fractogel TSK HW-40 (S) gel (Tosoh Corporation, Japan) in an aqueous solution of 1% acetic acid at a flow rate of 0.5 mL · min^−1^. Elution was monitored with a differential refractometer (Knauer, Germany).

### Animal immunization.

Animals were used according to the protocol “Evaluation of the protective effect of a number of glycoconjugates based on fragments of the capsular polysaccharide of the bacterial antigen Acinetobacter baumannii with proteins in BALB/c mice”, registration number 852/21 of 10.12.2021, approved at a meeting of the Institute’s Animal Care and Use Committee of BIBCh RAS on 17 December 2021. Animals were obtained from the Laboratory-Animal Breeding Nursery, Pushchino Branch, Institute of Bioorganic Chemistry, Russian Academy of Sciences, which has earned the international AAALACi accreditation. Experimental groups of 10 BALB/с females each were used for immunization with each preparation and each dose (50 or 100 μg).

Before immunization, the antigens were dissolved in phosphate-buffered saline (PBS). Then, an equal volume of Freund's adjuvant was added, and the contents were mixed intensively to form a stable water-oil emulsion (total volume, 200 μL/mouse). Thus, the samples that were administered to the animals included glycoconjugate, PBS, and adjuvant.

Immunization was performed at 2-week intervals. The first injection was performed using complete Freund’s adjuvant, and the following injections were performed using incomplete Freund’s adjuvant (Sigma, USA). The first injection was made subcutaneously into the paw pads, the second was made subcutaneously into the back, distributing the solution across as many points as possible, and the third was made in the same manner, distributing the contents across the abdominal surface of the body.

### Enzyme immunosorbent assay.

An indirect solid-phase EIA was used to determine the specific antibodies in the blood sera of the immunized animals. Purified A. baumannii CPSs were adsorbed on the surface of wells of high-binding EIA immune plates (Corning Costar, 2481, USA) from a 1 μg · mL^−1^ solution in 0.05 М carbonate buffer, рН 9.6, (50 μL) overnight at 4°С. Free binding center surfaces of the plastic were blocked by phosphate-buffered saline containing 0.1% Tween 20 (PBST) for 1 h. Then, immune sera 1:1000 diluted with PBS were added to the wells and titrated as a second step. The same diluted normal mouse serum was used as a negative-control. Incubation with the immobilized polysaccharides was performed at 37°C for 1 h. Then, the immune plate wells were washed with PBST no fewer than 6 times, and the conjugate of antibodies against mouse immunoglobulins was added (Corning Thermo Scientific 31432 Goat anti-Mouse IgG [H+L] HRP conjugate) in PBST, diluted as indicated by the manufacturer and incubated at 37°C for 1 h. A 4 mM solution of an ortho-phenylenediamine peroxidase substrate (Sigma, USA) in citrate phosphate buffer (26 mM citric acid, 50 mM Na_2_HPO_4_, рН 5.0) containing 0.003% (vol/vol) Н_2_O_2_ was added. After the development of the color, the reaction was stopped by adding an equal volume of 10% (vol/vol) sulfuric acid, and optical absorption was measured at 490 nm using an iMark microplate reader (Bio-Rad, USA).

For the determination of CPS-reacting IgM, goat antibodies against mouse IgM (Goat Anti-Mouse IgM H&L, ab9167, Abcam, USA) were added to experimental wells after incubation with the sera, and the mixture was incubated at 37°C for 1 h. Then, a conjugate of rabbit antibodies against goat immunoglobulins with peroxidase (P-RAG lss conjugate of rabbit antibodies to goat lgG, lgA, lgM with peroxidase [Imteck, Russia]) was added. Staining was performed as described earlier.

### Detection of subclass IgG1, IgG2a, and IgG2b immunoglobulins in serum.

An EIA was performed by sorbing on immune plates of goat anti-mouse IgG (Goat Anti-Mouse IgG H&L, 6708, Abcam, USA), followed by the titration of immune and nonimmune sera. After incubation and washing, rabbit antibodies against IgG subclasses (Bio-Rad, mouse typer isotyping panel, 1722055, USA) were added. Then, the mixture was incubated for 1 h and washed. Next, a conjugate of goat antibodies against rabbit immunoglobulins with peroxidase (Imteck, 110810, Russia) was added as recommended by the manufacturer. Staining was also carried out with an ortho-phenylenediamine solution. Wells without sera were used as negative-controls. Monoclonal antibodies of the IgG1, IgG2a, and IgG2b subclasses that were previously identified by this kit were used as calibrators and made into serial dilutions with steps of 2 starting at a concentration of 1 μg · mL^−1^ in wells with immobilized goat anti-mouse antibodies.

The determination of K9-binding IgG subclasses was performed similarly, but the K9 were immobilized on the immunoplate’s wells from a 1 μg · mL^−1^ solution. The serum and monoclonal antibodies’ preparations were measured in three repeats, each in a series of eight dilutions, starting from 1/100 for the serum of each mouse, separately. The contents of the IgG subclasses were calculated as described in (34, 52).

### The specificity of interaction in the EIA.

The specificity of interaction of the immune sera with the CPSs was determined via a competitive EIA. According to the titration curve, one dilution of each serum was selected so that the optical adsorption (А_490 nm_) on binding an immobilized polysaccharide would be in a linear region. In assay 1, the K9 CPS was absorbed from a 1 μg · mL^−1^ solution. The blocking of the binding centers and washing were performed as described earlier. As a second step, the polysaccharides were preliminarily titrated, starting from a concentration of 2 μg · mL^−1^, in PBST. The sera diluted with PBST were incubated separately with diluted solutions of the polysaccharides (AYE, AB5001, KZ-1093, KZ-1098, deacylated AB5001, and deacylated KZ-1098) at 37°C for 1 h.

After incubation, the mixtures were transferred into the wells of an EIA immunoplate with the immobilized K9 CPS, which were treated preliminarily with PBST for 1 h. The subsequent procedures were performed as described above. The nonimmune serum was diluted as the immune serum was added to the experimental wells, with the adsorbed polysaccharide serving as a negative-control. Each of the serum samples and inhibitor preparations was measured in three repeats with 8 dilutions in each series. The percentage of inhibition was calculated as follows: inhibition % = (A_490 nm_ + inhibitor/A_490 nm_ – inhibitor) ×100%.

In assay 2, the AYE, AB5001, KZ-1093, KZ-1098, deacylated AB5001, and deacylated KZ-1098 polysaccharides were adsorbed on plastic from 1 μg · mL^−1^ solutions. The blocking of the binding centers and washing were performed as described earlier. As a second step, the polysaccharides were preliminarily titrated, starting from a concentration of 60 μg · mL^−1^, in PBST. The sera were diluted with PBST and incubated separately with each dilution of the above polysaccharides at 37°C for 1 h.

After incubation, the mixtures were transferred into the wells of an EIA immune plate with PBST-treated, immobilized CPSs. The incubation mixtures of the diluted sera with AYE were transferred to the wells with adsorbed AYE, the mixtures of sera with AB5001 were transferred to the wells with adsorbed AB5001, and so on. The subsequent experiments and the calculation of the inhibition % were performed as described above.

### Opsonization assay.

The opsonization ability of the sera of the immunized mice was studied with respect to various A. baumannii strains, using a modified method of (53).

A. baumannii bacteria were grown in a 2YT medium (16 g · L^−1^ bactotryptone, 1 g · L^−1^ yeast extract, 5 g · L^−1^ NaCl, рН 7.0) overnight. The infected exponential culture was cultivated at 28°C and 200 rpm for 2 h. The CFU concentration was determined by measuring the optical density at 600 nm, and the concentration of bacteria was measured against the preliminary experiments on the titrations of the bacteria. A suspension of A. baumannii bacteria in the exponential growth phase was mixed with glycerol (1:1) and frozen at –70°C in 50 μL aliquots. Before the experiments, control seeding was done from one aliquot to determine the concentration of the CFU. Thus, a standardized suspension of A. baumannii bacteria was used for all experiments. The opsonization ability was measured in the presence of a complement (normal rabbit serum). The optimal amount of the complement was determined in preliminary experiments. The experimental and control (nonimmune) sera were preliminary inactivated at 56°C for 30 min.

250 CFU of A. baumannii and diluted serum of an immunized mouse were added into each experimental point. The correspondingly diluted nonimmune mouse serum was used as a negative-control for each point. In addition, complement (5 μL of normal rabbit serum) and bovine serum albumin were added up to a concentration of 3 μg · mL^−1^. Then, phosphate-buffered saline was added up to a volume of 100 μL. The obtained mixture was incubated at 37°C for 1 h under stirring. Prior to the experiment, J774 macrophages (CLS Cell Lines Service, 440220GR, mouse monocytes, macrophage) were inoculated into 96-well culture plates (5 × 10^4^ per well) and cultivated overnight in a wet atmosphere containing 5% CO_2_ at 56°C. The wells were washed with PBS to eliminate the supernatants. The experimental mixtures were then added, and after centrifugation (1,000 rpm, 5 min), they were incubated for 1 h in a wet atmosphere containing 5% CO_2_ at 37°C. The final ratio of bacteria to macrophage cells was 1:200. A 10 μL aliquot of untouched bacteria was diluted 4-fold and 8-fold and then inoculated onto the mixture of a 2YT medium and 1.5% agar. Bacteria were grown overnight at 37°C, and the number of colonies was calculated on the next day.

### Preparation of the splenocyte suspension and the сell viability test.

Spleens were collected immediately after euthanasia and separated into sterile plastic petri dishes (7 × 1.5 cm, Greiner, Germany), each containing PBS. Splenocytes were isolated according to the method of (54).

The viability of peripheral blood mononuclear cells, as well as that of isolated splenocytes, was tested by 0.02% trypan blue exclusion and light microscopy (55), based on the impermeability of viable cells to trypan blue. The percentage of viable lymphocytes/mL was calculated by the following equation: % of viable cells = (number of viable cells/total number counted) × 100.

### Analysis of the lymphokine profile after glycoconjugate immunization and CPS stimulation *in vitro*.

The animals (*n *= 10 BALB/c mice per group and *n *= 10 ICR mice per group) received two intraperitoneal injections (100 μg glycoconjugate no. 10 in 200 μL PBS) at 21-day intervals. On day 26, spleens were aseptically removed from all animals in all groups, and splenocytes were suspended in a DMEM medium with 10% fetal bovine serum and placed into the wells of 24-well culture plates (2 × 10^6^ cells/well). In parallel, splenocytes were also obtained from intact mice (*n *= 10 mice per group) without prior immunization with glycoconjugate. Various amounts of K9 CPS solution (1, 10, and 100 μg) were added to the cells in 3 independent repeats, and these served as the sources of the carbohydrate moiety during glycoconjugate synthesis. The plates were incubated for 48 h, after which the contents of IL-4, IL-10, IL-17A, γ-IFN, and TNF-α were analyzed using BD Cytometric Bead Array flex set beads (mouse IL-4 [cat. no 558298], mouse IL-10 [cat. no 558300], mouse IL-17A [cat. no 560283], mouse γ-IFN [cat. no 558296], and mouse TNF-α [Cat. No 558299]) according to the manufacturer's instructions. The measurements were performed using a NovoCyte Advanteon flow cytometer (Agilent, USA).

### Survival against a challenge with A. baumannii.

To determine whether glycoconjugate immunization could protect mice from an A. baumannii K9 infection, BALB/c mice immunized according to the above scheme with glycoconjugate no. 12 with Freund's adjuvant (20 individuals) and with calcium phosphate as an adjuvant (20 individuals) were subjected to a bacterial challenge. Control mice received either only Freund's adjuvant injections (20 mice), or only calcium phosphate (20 mice). The calcium phosphate adjuvant was obtained according to (37). For this, equal volumes of 14 mM Na_2_SO_4_ and CaCl_2_ solutions were mixed on a magnetic stirrer. Then, the NaOH was neutralized, glycoconjugate no. 12 was added, and the mixture was stirred for 6 h.

The strain A. baumannii K9 was cultivated overnight at 37°C under stirring in a 2YT medium and then cultivated under the same conditions until the middle of the logarithmic growth phase. The bacteria (lethal dose, 10^8^ CFU per mouse) were washed twice with PBS by centrifugation at 1,500 × *g* in 200 μL PBS. The concentration of A. baumannii was confirmed by quantitative cultivation in a mixture of a 2YT medium and 1.5% agar. The immunized mice were infected intraperitoneally by injection of the inoculate on day 14 after the end of the immunization cycle. At 10 h after the infection, groups of 5 animals (immunized and nonimmunized) each were killed, and the blood and spleen of each animal was taken for analysis. The spleens were taken under sterile conditions and homogenized in sterile PBS. The spleen homogenates and the blood preparations of the individual mice were cultivated in a mixture of a 2YT medium and 1.5% agar to determine the bacterial burden.

### Statistical analysis.

The analysis was performed using Microsoft Excel and OriginPro8 software. An unpaired two-tailed Student's *t* test or a Mann–Whitney test was used to analyze the statistical significance between the intact and experimental groups as well as to compare the experimental groups with one another. The method of statistical analysis was based on the Shapiro-Wilk normality test. Statistical significance was achieved at *P*  <  0.05. Data are presented as means ± standard errors of the means (SEM).
